# The Canadian multi‐ethnic research on aging (CAMERA) study: Design, participant characteristics, and preliminary findings

**DOI:** 10.1002/alz.71344

**Published:** 2026-04-14

**Authors:** Tulip Marawi, Harleen Rai, Rohina Kumar, Katie L. Vandeloo, Rachel Yep, Madeline Wood Alexander, Silina Z. Boshmaf, Sarah‐Mei Chen, Georgia Gopinath, Simran Malhotra, Angelina Zhang, Alexander J. Nyman, Lucas Xavier Perri, Valery Sit, Tallinn F. L. Splinter, Douglas P. Munoz, Walter Swardfager, Jennifer D. Ryan, Sandra E. Black, Maged Goubran, Jennifer S. Rabin

**Affiliations:** ^1^ Hurvitz Brain Sciences Program Sunnybrook Research Institute Toronto ON Canada; ^2^ Graduate Department of Psychological Clinical Science University of Toronto Scarborough Toronto ON Canada; ^3^ Rehabilitation Sciences Institute University of Toronto Toronto ON Canada; ^4^ Department of Biomedical & Molecular Sciences Queen's University Kingston Canada; ^5^ The Centre for Neuroscience Studies, Kingston Health Sciences Centre; Division of Neurology, Department of Medicine Queen's University Kingston Canada; ^6^ Department of Pharmacology & Toxicology University of Toronto Toronto ON Canada; ^7^ Rotman Research Institute Baycrest Health Sciences Toronto ON Canada; ^8^ Division of Neurology, Department of Medicine, Sunnybrook Health Sciences Centre University of Toronto Toronto ON Canada; ^9^ Harquail Centre for Neuromodulation Sunnybrook Research Institute Toronto ON Canada; ^10^ Department of Medical Biophysics University of Toronto Toronto ON Canada

**Keywords:** ADRD, Alzheimer's disease, Asian, Chinese, dementia, ethnoracial diversity, South Asian

## Abstract

**INTRODUCTION:**

South Asian and Chinese individuals are the largest and fastest‐growing ethnoracial groups in Canada, yet they remain underrepresented in dementia research. To address this gap, we established the CAnadian Multi‐Ethnic Research on Aging (CAMERA) study.

**METHODS:**

CAMERA is a longitudinal observational study conducted in Toronto, Canada, enrolling 300 adults aged 55‐85 who self‐identify as South Asian, Chinese, or non‐Hispanic White (NHW). Participants complete in‐person visits at baseline, Year 3, and Year 5, which include clinical and cognitive assessments, brain magnetic resonance imaging (MRI), and blood‐based biomarkers. Annual remote questionnaires track health and lifestyle factors.

**RESULTS:**

Among the first 200 participants, vascular and metabolic profiles differed across groups. In addition, South Asian and Chinese participants reported greater cognitive concerns than NHW participants and had lower Montreal Cognitive Assessment (MoCA) scores. The latter was driven primarily by language‐heavy and culturally dependent items. Eye‐tracking measures did not differ across groups.

**DISCUSSION:**

CAMERA provides a deep phenotyping framework to investigate dementia risk and resilience factors in Asian Canadians.

## Highlights

1. South Asian and Chinese Canadians are rapidly growing populations but remain underrepresented in Alzheimer's disease and related dementias (ADRD) research.
2. CAnadian Multi‐Ethnic Research on Aging (CAMERA) is the first longitudinal deep phenotyping study of ADRD risk and resilience factors in Asian Canadians.
3. South Asian participants showed lower high‐density lipoprotein cholesterol, higher triglycerides, and elevated HbA1c levels compared to non‐Hispanic White (NHW) participants. Chinese participants exhibited higher HbA1c levels compared to NHW participants, despite lower body mass index.
4. South Asian and Chinese participants endorsed greater cognitive concerns than NHW participants and had lower Montreal Cognitive Assessment (MoCA) scores. The latter was driven by items with greater reliance on English proficiency and Western cultural knowledge.

## 1 BACKGROUND

Alzheimer's disease (AD) and related dementias (ADRD) represent a major and growing public health challenge, with substantial personal, societal, and economic impacts.^1^ Despite their increasing burden, much of the existing research in North America has been conducted in predominantly non‐Hispanic White (NHW) cohorts. This focus has limited our understanding of how ADRD risk and resilience factors differ across racial and ethnic populations,^2^, ^3^, ^4^, ^5^, ^6^ which is critical for developing effective and equitable prevention and intervention strategies.

This knowledge gap is especially pronounced among individuals of Asian descent.^2^, ^3^, ^7^, ^8^ In Canada, studies of aging and ADRD report low levels of Asian representation^9^, ^10^ relative to their population share (approximately 20%).^11^ Comparable patterns of underrepresentation are evident in the U.S. For example, in the National Alzheimer's Coordinating Center (NACC) Uniform Data Set, only 2.6% of participants are of Asian descent, despite Asian individuals comprising nearly 14% of older adults in the U.S.^12^, ^13^

Beyond underrepresentation, a major limitation of the existing literature is the routine aggregation of heterogeneous Asian subpopulations into a single, monolithic “Asian” category.^14^ This limitation is especially consequential for South Asian and Chinese populations, which are the two largest and fastest‐growing Asian groups in Canada.^11^ Treating these groups as homogeneous obscures meaningful between‐group differences across a range of clinically relevant factors, including vascular risk burden, a well‐established contributor to dementia.^15^, ^16^

Evidence from Canadian and U.S. studies consistently demonstrates substantial heterogeneity in vascular risk profiles across Asian subpopulations.^17^, ^18^, ^19^ Specifically, South Asian individuals tend to exhibit a higher vascular risk burden than both Chinese and NHW individuals, with elevated risk often emerging at younger ages. In contrast, Chinese individuals generally have a lower vascular risk burden than these groups.^17^, ^18^, ^19^, ^20^, ^21^ However, some evidence suggests that Chinese immigrant populations may experience a higher vascular risk burden compared to individuals living in China, with risk increasing with longer duration of residence in the host country.^22^ Although epidemiological data on dementia incidence among Asian subgroups remain limited, available evidence aligns with these patterns. For example, U.S. data indicate that South Asian individuals may a higher incidence of dementia than Chinese individuals and other ethnoracial groups,^23^ with these differences particularly pronounced among South Asian individuals with diabetes.^24^

Given this heterogeneity, there is a clear need for longitudinal research to characterize ADRD risk and resilience in Asian subgroups. Subgroup‐specific insights are critical, as differences in vascular burden, metabolic profiles, and sociocultural factors may influence disease trajectories and inform prevention and intervention efforts. Because ADRDs develop gradually over decades, with neuropathology emerging long before symptoms appear, biomarker‐informed longitudinal cohorts provide a powerful way to detect early biological changes and assess whether disease pathways differ across ethnoracial groups.

Canada is particularly well positioned to support this work. South Asian (i.e., Indian, Pakistani, Sri Lankan, Bangladeshi) and Chinese communities together comprise a substantial and growing proportion of the Canadian population, accounting for 7.1% and 4.7% of Canadians, respectively.^11^ In major urban centers, such as Toronto, these proportions are even higher, with South Asian and Chinese communities comprising 14% and 10.7% of the population.^11^ This demographic context provides an important opportunity to conduct longitudinal, biomarker‐informed research in groups that have been historically underrepresented in ADRD studies.

To leverage this opportunity, we established the CAnadian Multi‐Ethnic Research on Aging (CAMERA) study, a longitudinal observational cohort based in Toronto that investigates mechanisms underlying ADRD risk and resilience factors among underrepresented South Asian and Chinese populations. CAMERA employs a deep phenotyping approach, with participants completing comprehensive biannual assessments, including clinical evaluations, brain imaging, blood‐based biomarker assessments, questionnaires, and both standard and novel cognitive tests designed to minimize language demands and cultural influences. By integrating biomarker, clinical, and cognitive data, CAMERA aims to advance understanding of population‐specific biological pathways and inform the development of ADRD prevention and intervention strategies.

## 2 METHODS

The CAMERA study was approved by the Research Ethics Board at Sunnybrook Health Sciences Centre in Toronto, Canada.

### 2.1 Participants

The CAMERA study is an ongoing longitudinal cohort that aims to recruit and retain 300 community‐dwelling adults aged 55 to 85 years who self‐identify as South Asian, Chinese, or NHW (100 participants per group).

Recent Canadian census data indicate that approximately 71% of South Asian and 56% of Chinese adults aged 65 and older can converse in English.^25^ Given this level of language proficiency, along with the practical and resource considerations involved in validating and administering assessments in multiple languages, all study assessments are conducted in English. However, to support participants and create a welcoming testing environment, language assistance is available in Punjabi, Hindi, Urdu, Cantonese, and Mandarin.

**RESEARCH IN CONTEXT**

1. **Systematic review**: We reviewed the existing literature on Alzheimer's disease and related dementias (ADRD) across Asian subgroups. Despite being among the largest and fastest‐growing ethnoracial groups in Canada, South Asian and Chinese populations remain markedly underrepresented in ADRD research. Emerging evidence suggests potential differences in dementia incidence between these groups, possibly shaped by modifiable risk and resilience factors. However, these relationships remain poorly characterized and incompletely understood.
2. **Interpretation**: To address this gap, we established the CAnadian Multi‐Ethnic Research on Aging (CAMERA) study, an ongoing longitudinal observational cohort that uses a deep phenotyping approach to examine risk and resilience factors among South Asian and Chinese Canadians. A Community Advisory Board guides study implementation and dissemination to promote cultural relevance and sustained community engagement. Here, we report clinical, cognitive, and neuroimaging data from the first 200 participants.
3. **Future directions**: CAMERA aims to expand its cohort and extend follow‐up beyond 5 years, enabling a deeper understanding of long‐term ADRD risk and resilience trajectories in these underrepresented populations.

Eligibility criteria for the CAMERA study include: (1) self‐identification as South Asian, Chinese, or NHW; (2) sufficient English proficiency to provide informed consent and complete study assessments. This was assessed at screening using a 5‐point Likert scale (1 = none, 3 = adequate, 5 = proficient) for speaking, understanding, and reading English. Participants are required to score ≥3 in all domains, with proficiency additionally confirmed by the study screener; (3) a minimum of 8 years of formal education to support meaningful participation in study assessments and questionnaires, which are written at a grade 6–8 reading level; (4) a global Clinical Dementia Rating (CDR)^26^ of 0 (clinically normal) or 0.5 (mild cognitive difficulties) at baseline; (5) adequate (or corrected) vision and hearing to participate in cognitive testing; and (6) availability of a study partner who knows the participant well, maintains at least weekly contact, and can provide informed observations about their cognition and daily functioning.

Exclusion criteria were designed to minimize confounding factors and to exclude conditions that could obscure associations of interest, including: (1) history of major cardiovascular or cerebrovascular events (e.g., myocardial infarction, symptomatic stroke); (2) presence of unstable medical conditions (e.g., pulmonary or endocrine disorders); (3) active infectious diseases; (4) current diagnosis of major psychiatric disorders, neurological disorders, or dementia; (5) history of a significant learning disability; (6) history of significant head trauma or recurrent concussions with loss of consciousness or hospitalization; (7) pain or sleep disorders that could interfere with cognitive testing; (8) recent history of substance or drug abuse; (9) medical conditions that may obscure relevant relationships (e.g., active cancer); and (10) magnetic resonance imaging (MRI) contraindications.

### 2.2 Study design

**FIGURE 1:**
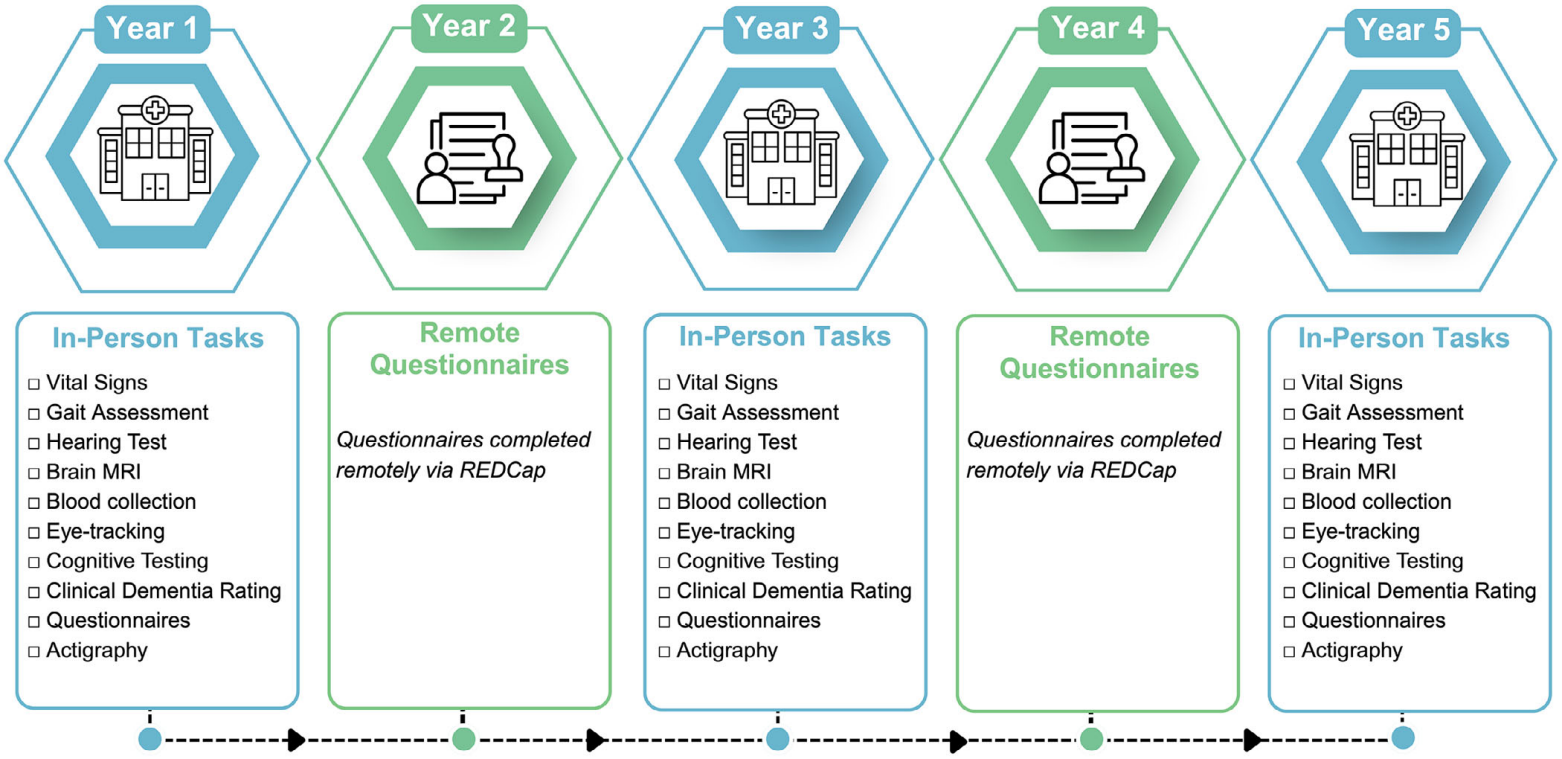
Timeline and assessments. The figure depicts the study timeline and procedures of the CAMERA study across the 5‐year study period. Participants complete in‐person assessments at baseline (Year 1), Year 3, and Year 5, and complete questionnaires annually on REDCap. CAMERA, the CAnadian Multi‐Ethnic Research on Aging.

Participants complete in‐person assessments at baseline (Year 1), Year 3, and Year 5. Each in‐person assessment involves two study visits scheduled within a 3‐month window. These visits involve comprehensive clinical assessments (vital signs, anthropometric measures, gait assessment, hearing assessment), brain MRI, a blood draw, cognitive testing (including eye tracking tasks), as well as questionnaires. Questionnaires can be completed on‐site or remotely via REDCap. At the end of the first visit, participants are provided with an actigraphy device to wear for 12 consecutive days to monitor physical activity and sleep patterns. During the intervening years (Years 2 and 4), participants complete questionnaires remotely via REDCap (Figure 1).

### 2.3 Study measures

#### 2.3.1 Clinical assessments

##### 2.3.1.1 CDR

Participants and their study partners complete the CDR,^26^ which is administered by trained personnel at baseline (Year 1), Year 3, and Year 5.

##### 2.3.1.2 Vital signs

Blood pressure and heart rate are measured using an automated sphygmomanometer. Six readings (three seated and three standing) are taken 1 minute apart. Respiratory rate, waist circumference, hip circumference, height, and weight are also collected.

##### 2.3.1.3 Gait assessment

Gait is measured using a pressure‐sensitive walkway (GaitRite)^27^ that records the timing and placement of each step using protocols from the Ontario Neurodegenerative Disease Research Initiative (ONDRI).^9^, ^28^ Gait performance is assessed under both single‐task (preferred and fast walking) and dual‐task conditions. There are three dual‐tasks: (1) counting backward from 100 by ones; (2) counting backward from 100 by sevens; and (3) naming as many different animals as possible. These cognitive tasks are included solely to create a dual‐task condition and are not scored. All gait assessments take place after neuropsychological testing to avoid practice effects with similar tests.

##### 2.3.1.4 Hearing test

Participants complete a hearing assessment using the Triple Digit Test.^29^, ^30^ In this task, participants listen to a voice recording in which three digits are presented against different levels of background noise and are asked to report the digits in the correct order.

### 2.4 Brain MRI

Brain MRI scans are acquired on a 3T Siemens MAGNETOM Prisma magnetic resonance system, using a 64‐channel head coil. Each 50‐minute scanning session includes the following sequences (see Table S1 for more details): (1) three‐dimensional T1‐weighted magnetization prepared rapid gradient echo (MPRAGE) acquired in the sagittal plane; (2) three‐dimensional T2‐weighted Sampling Perfection with Application optimized Contrast using different flip angle Evolution (SPACE) acquired in the axial plane; (3) 3‐dimensional T2‐weighted fluid‐attenuated inversion recovery (FLAIR) acquired in the sagittal plane; (4) resting‐state functional MRI (rs‐fMRI) acquired in two phase‐encoding (PE) directions (anterior‐to‐posterior, and posterior‐to‐anterior); (5) multi‐shell diffusion weighted imaging (DWI) acquired with 95 diffusion directions in two PE directions (anterior to posterior (*b* = 2000s/mm^2^, *b* = 1000s/mm^2^) and posterior‐to‐ anterior (*b* = 0s/mm^2^) for distortion correction); (6) a high‐resolution T2‐weighted turbo spin echo (TSE) hippocampal scan, oblique to the long axis of the hippocampus; (7) pseudo‐continuous arterial spin labeling (ASL); and (8) susceptibility weighted imaging (SWI).

### 2.5 Cognitive assessments

#### 2.5.1 Eye‐tracking

A major barrier to conducting ADRD research in diverse ethnoracial groups is that most cognitive tests were developed and validated in English‐speaking NHW individuals born in Anglosphere countries.^31^ As a result, their validity may be compromised when administered to adults from different linguistic and cultural backgrounds.^31^, ^32^ Strategies to address this limitation include translating existing tests, developing group‐specific norms, and/or using an interpreter to conduct neuropsychological testing.^33^, ^34^ However, these approaches are resource‐intensive and may not fully eliminate bias. An alternative approach employed in the CAMERA study is to incorporate tests that minimize language demands (beyond the instructions themselves) and reduce cultural influences. As part of this approach, we include eye‐tracking tasks, a widely accepted method for assessing cognition that has been applied in non‐human primates,^35^, ^36^, ^37^ infants,^38^, ^39^, ^40^ and adults with aphasia or speech limitations.^41^, ^42^ Eye‐tracking tasks are particularly advantageous because they can be designed to minimize language‐dependent and culturally biased stimuli and do not require verbal responses, making them especially well‐suited for participants from diverse linguistic backgrounds.

The CAMERA study employs two well‐validated eye‐tracking tasks: the Interleaved Pro/Anti‐Saccade Task (IPAST)^43^, ^44^ and the Visual Paired Comparison Task (VPCT).^45^ The IPAST assesses processing speed and executive function, whereas the VPCT assesses episodic memory.

**FIGURE 2:**
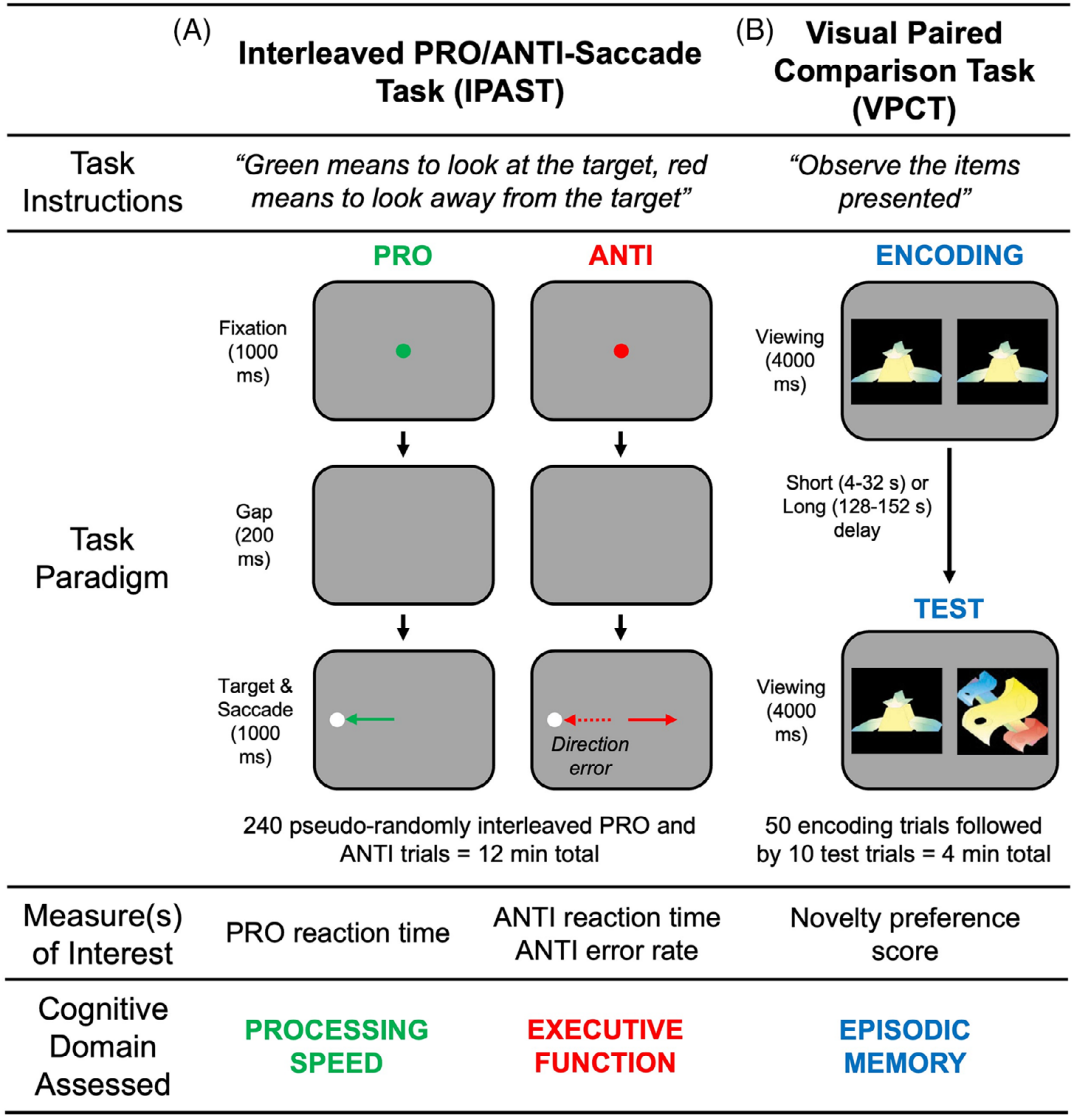
Eye tracking measures. (A) The Interleaved PRO and ANTI Saccade Task (IPAST). (B) The Visual Paired Comparison Task (VPCT).

In the IPAST, participants complete intermixed pro‐ and anti‐saccade trials, in which they are instructed either to look toward a peripheral visual stimulus (pro‐saccade) or to inhibit this response and look in the opposite direction (anti‐saccade)^43^, ^44^ (see Figure 2A). Saccadic reaction time on pro‐saccade trials provides a measure of processing speed through sensorimotor pathways, whereas saccadic reaction time and direction errors on anti‐saccade trials provide measures of executive function.^43^, ^44^, ^46^ Previous studies demonstrate that both pro‐ and anti‐saccade performance declines with age^47^, ^48^, ^49^, and that anti‐saccade performance is impaired in mild cognitive impairment and dementia.^50^, ^51^

The VPCT begins with a familiarization phase in which participants are presented with pairs of identical abstract images^45^ (see Figure 2B). In the test phase, participants are presented with a new series of image pairs, each consisting of one “old” image from the familiarization phase and one “novel” image not previously seen.^45^ Based on the well‐established novelty effect,^52^, ^53^ participants with intact memory are expected to spend more time viewing the novel image than the old image (i.e., novelty preference). Prior work shows that novelty preference on the VPCT declines with age,^54^ is impaired in mild cognitive impairment,^52^ and predicts progression to AD dementia.^55^, ^56^

#### 2.5.2 Standard neuropsychological tests

In addition to eye‐tracking tasks, participants complete a battery of standardized neuropsychological tests assessing processing speed, executive function, episodic memory, language, and visuospatial abilities (see Table 1). The battery includes both paper‐ and computer‐based assessments and was designed to minimize language demands and culturally specific stimuli. However, a subset of commonly used language‐based measures is included to facilitate comparison with existing literature, despite the potential for cultural and linguistic bias in these assessments.

**TABLE 1 alz71344-tbl-0001:** CAMERA neuropsychological assessment battery.

Cognitive domain	Tests
Global cognition	Montreal Cognitive Assessment (MoCA) Clinical Dementia Rating (CDR) scale
Processing speed	Digit symbol‐coding subtest from WAIS‐III Trail‐Making Test Part A Color Trails Test I
Executive function	N‐Back Task (1‐Back and 2‐Back) Sustained Attention to Response Task (SART) Trail‐Making Test Part B Color Trails Test II Phonemic Fluency (FAS)
Visuoperceptual/visuospatial processing	Four Choice Oddity Task Benson Complex Figure Test—Copy Trial
Learning and memory	Free and Cued Selective Reminding Test (Form A) 7/24 Learning, Immediate Recall, and Delayed Recall (Form I) Benson Complex Figure Test—Delayed
Language	Category Fluency (Animals, Fruits, Vegetables)
Pattern separation	Doors and People Test (Doors Subtest) Mnemonic Similarity Task (MST—Set C)

Abbreviation: WAIS‐III, Wechsler Adult Intelligence Scale—Third Edition.

### 2.6 Questionnaires

Participants complete a series of questionnaires annually via REDCap (see Table S2). The questionnaires capture a range of variables, including: (1) demographic information; (2) socioeconomic status; (3) immigration history; (4) linguistic background; (5) acculturation; (6) environmental and community factors; (7) medical history; (8) mental health; (9) physical activity and sedentary behavior; (10) sleep quality; (11) quality of life; (12) perceived discrimination; (13) cognitive concerns; (14) functional status; and (15) sex‐specific health history.[Table alz71344-tbl-0001]

#### 2.6.1 Actigraphy

Participants are provided with a wearable wrist accelerometer (GENEActive) that continuously records daytime and nighttime activity. Participants are asked to wear the accelerometer for 12 consecutive days.^57^

### 2.7 Blood collection

Participants undergo a fasting venous blood draw in the morning. Blood samples are processed at Sunnybrook Health Sciences Centre and analyzed to measure lipids (i.e., total cholesterol, low‐density lipoprotein [LDL], high‐density lipoprotein [HDL], and triglycerides), glucose regulation (i.e., hemoglobin A1c, HbA1c), and inflammation (i.e., C‐reactive protein). Serum, plasma, and white blood cell samples are also stored for future analyses, including apolipoprotein E (*APOE*) ε4 genotyping, as well as markers of AD and neurodegeneration, and more detailed inflammatory profiling.

### 2.8 Community‐based participatory research approaches

The Community Advisory Board (CAB) for the CAMERA study was established in January 2024 to ensure that study logistics, including implementation, assessments, and dissemination, are guided by community perspectives. Members were recruited through partnerships with local community organizations, outreach events, and word‐of‐mouth referrals. The CAB currently includes six community members whose identities and lived experiences reflect those of the CAMERA cohort. The CAB includes two South Asian members, three Chinese members, and one NHW member (mean age = 61.3 years, mean years of education = 19 years). Most members also have lived experience with dementia as care partners. Several members later chose to enroll as study participants, providing firsthand experience with study procedures and enhancing the relevance of their feedback. Additional demographic characteristics of the CAB members are provided in Table S3.

The CAB meets quarterly, with additional ad hoc meetings as needed, providing ongoing input across the study lifecycle. This includes aligning research objectives with community priorities, supporting community outreach and recruitment, reviewing study materials for cultural appropriateness, and co‐developing accessible strategies for knowledge dissemination.

#### 2.8.1 Participation benefits

A core value of the CAMERA study is a strong commitment to sharing individualized results with participants. Sharing results promotes transparency, builds trust, and acknowledges participants as valued contributors to the research.^58^, ^59^ Participants receive feedback in four domains: (1) *MRI results*: Participants may request access to their MRI data and radiology report. If incidental findings are identified, results are communicated to participants, and referrals are facilitated. (2) *Cognitive testing*: Participants may request a feedback session with a neuropsychologist (JSR) to review their cognitive results. If testing identifies previously unrecognized cognitive impairment, participants are informed and referred to a specialist. (3) *Blood test results*: Participants may request results from the standard blood panel (e.g., lipid profile and glucose measures). Clinically relevant findings are reviewed with the participant, and shared with the participant's primary care physician. (4) *Mental health results*: When questionnaire responses indicate elevated symptoms of depression or anxiety, participants are informed and encouraged to discuss these findings with their primary care physician. Participants are also offered relevant resources, including information on free or low‐cost, culturally appropriate counselling services.

### 2.9 Recruitment and retention strategies

We adopted a community‐informed approach to recruitment designed to engage participants from all three ethnoracial groups. This approach includes community‐based presentations on dementia risk factors, during which the CAMERA study is introduced and attendees are invited to participate. Additional recruitment efforts focus on culturally tailored outreach within South Asian and Chinese communities in the Greater Toronto Area. This includes targeted advertising in senior and community centers, newsletters, and websites serving South Asian and Chinese communities, and outreach through social media. Word‐of‐mouth referrals have also greatly supported enrollment across all three groups, reflecting participants’ positive experiences with the study.

To support retention and foster a sense of engagement, we emphasize participant‐centered practices throughout the study. These include sharing individualized results, providing resources and facilitating referrals when incidental findings arise, and compensating participants for their time and effort. To maintain ongoing connection with participants, we send quarterly newsletters that include study updates, highlights from recent research, and educational content related to brain health.

## 3 RESULTS

### 3.1 Participants characteristics

Recruitment for the CAMERA study began in mid 2022 and is ongoing. The current analysis includes participants enrolled through July 2025. Baseline demographic and clinical characteristics are summarized in Table 2. As of July 2025, 200 participants had completed baseline (Year 1) assessments, including 52 South Asian participants (42 Indian, three Pakistani, two Bangladeshi, and five unspecified), 77 Chinese participants, and 71 NHW participants. Among Chinese participants, self‐identified subgroups included 19 Chinese (e.g., Zhuang, Hui, Manchu), 17 Han Chinese, and 24 Hong Kongers. An additional 10 participants selected more than one subgroup (four Chinese and Han Chinese; three Han Chinese and Hong Konger; two Chinese and Hong Konger; one selected all three), and seven did not specify a subgroup.

**TABLE 2:** Baseline demographic, cognitive, and clinical characteristics.

Variable	Total (*n* = 200)	South Asian (*n* = 52)	Chinese (*n* = 77)	NHW (*n* = 71)	Omnibus *p*	Post‐hoc
**Demographic characteristics**						
Age (years), range	67.3 ± 6.9, 55‐84	68.1 ± 7.6, 55‐84	68.0 ± 5.5, 58‐81	65.8 ± 7.4, 55‐82	0.06	NA
Sex (female), *n* (%)	136 (68.0)	38 (73.1)	47 (61.0)	51 (71.8)	0.25	NA
Education (years)	16.5 ± 2.3	16.9 ± 2.3	16.2 ± 2.1	16.6 ± 2.4	0.21	NA
**Annual income, CAD*, n* (%)^a^**						
<$10,000–$49,999	18 (9.0)	2 (3.8)	12 (15.8)	4 (5.6)	0.17	NA
$50,000–$149,999	71 (35.7)	17 (32.7)	30 (39.5)	24 (33.8)		
$150,000+	80 (40.2)	23 (44.2)	26 (34.2)	31 (43.7)		
Prefer not to disclose	30 (15.1)	10 (19.2)	8 (10.5)	12 (16.9)		
**Immigration variables**						
Born in Canada, *n* (%)^a^	64 (32.2)	1 (1.9)	10 (13.2)	53 (74.6)	<0.001	SA < NHW, *p* < 0.001 Chinese < NHW, *p* < 0.001 SA vs. Chinese, *p* = 0.06
Age at immigration^*^	26.6 ± 12.5	24.3 ± 9.2	29.9 ± 13.1	21.6 ± 15.8	0.02	SA vs. NHW, *p* = 0.77 Chinese vs. NHW, *p* = 0.12 Chinese > SA, *p* = 0.02
Years since immigration^*^ ^b^	41.0 ± 13.2	43.7 ± 9.6	37.7 ± 13.8	45.4 ± 17.3	0.02	SA vs. NHW, *p* = 0.91 Chinese vs. NHW, *p* = 0.21 Chinese < SA, *p* = 0.02
English as second language, n (%)	125 (62.5)	40 (76.9)	68 (88.3)	17 (23.9)	< 0.001	SA > NHW, *p* < 0.001 Chinese > NHW, *p* < 0.001 Chinese vs. SA, *p* = 0.14
**Clinical characteristics (adjusted for age, sex, and years of education)**						
GDS^b^	4.8 ± 4.8	4.8 ± 5.4	4.6 ± 4.1	5.0 ± 5.0	0.90	NA
GAD‐7^b^	2.1 ± 3.1	2.3 ± 3.3	2.3 ± 3.5	1.7 ± 2.2	0.27	NA
WHOQOL‐OLD^b^	94.6 ± 11.8	93.5 ± 13.2	92.1 ± 10.8	98.0 ± 11.2	0.01	SA < NHW, *p* = 0.04 Chinese < NHW, *p* = 0.005 Chinese vs. SA, *p* = 0.57
HbA1c (%)^b^	5.8 ± 0.5	5.8 ± 0.5	6.0 ± 0.5	5.5 ± 0.3	0.001	SA > NHW, *p* < 0.001 Chinese > NHW, *p* < 0.001 SA vs. Chinese, *p* = 0.11
Total cholesterol (mmol/L)^d^	5.2 ± 1.2	5.0 ± 1.3	5.1 ± 1.3	5.3 ± 1.1	0.46	NA
HDL cholesterol (mmol/L)^d^	1.7 ± 0.5	1.6 ± 0.4	1.8 ± 0.5	1.8 ± 0.5	0.003	SA < NHW, *p* = 0.002 Chinese vs. NHW, *p* = 0.99 SA < Chinese, *p* = 0.002
LDL cholesterol (mmol/L)^d^	2.9 ± 1.0	2.9 ± 1.1	2.8 ± 1.1	3.1 ± 0.9	0.51	NA
Non‐HDL cholesterol (mmol/L)^d^	3.5 ± 1.1	3.5 ± 1.2	3.4 ± 1.2	3.5 ± 1.0	0.69	NA
Triglycerides (mmol/L)^d^	1.1 ± 0.6	1.4 ± 0.7	1.1 ± 0.6	1.0 ± 0.5	0.005	SA > NHW, *p* = 0.001 Chinese vs. NHW, *p* = 0.41 SA > Chinese, *p* = 0.01
Systolic BP^b^	131.7 ± 16	133.4 ± 13.2	131.9 ± 16.3	130.2 ± 17.5	0.64	NA
Diastolic BP^b^	73.9 ± 9.0	72.5 ± 8.9	74.8 ± 9.2	73.8 ± 8.8	0.63	NA
Waist‐to‐hip ratio^e^	0.9 ± 0.1	0.9 ± 0.1	0.9 ± 0.1	0.9 ± 0.1	0.51	NA
BMI, (kg/m^2^)^f^	25.17 ± 4.23	25.8 ± 3.9	23.8 ± 3.8	26.2 ± 4.6	0.001	SA vs. NHW, *p* = 0.66 Chinese < NHW, *p* < 0.001 Chinese < SA, *p* = 0.006
**Cognitive scores (adjusted for age, sex, and years of education)**						
CDR Global of 0, n (%)^c^	189 (97.4)	52 (100)	71 (94.7)	66 (98.5)	0.22	NA
CFI^b^	2.0 ± 2.1	2.1 ± 2.3	2.6 ± 2.2	1.2 ± 1.5	< 0.001	SA > NHW, *p* = 0.04 Chinese > NHW, *p* < 0.001 Chinese vs SA, p = 0.10
MoCA^a^	26.1 ± 2.9	25.2 ± 3.2	25.3 ± 2.8	27.4 ± 2.1	< 0.001	SA < NHW, *p* < 0.001 Chinese < NHW, *p* < 0.001 SA vs Chinese, *p* = 0.66

Abbreviations: ANOVA, analysis of variance; BMI, body mass index; BP, blood pressure; CAD, Canadian Dollar; CDR, Clinical Dementia Rating; CFI, Cognitive Function Index; GAD‐7, Generalized Anxiety Disorder–7; GDS, Geriatric Depression Scale; HbA1c, hemoglobin A1c; HDL, high‐density lipoprotein; LDL, low‐density lipoprotein; MoCA, Montreal Cognitive Assessment; NA, not applicable; NHW, non‐Hispanic White; SA, South Asian; SD, standard deviation; WHOQOL‐OLD, World Health Organization Quality of Life–Older Adults Module. Continuous variables are presented as mean ± SD. a, 1 missing value; b, 2 missing values; c, 6 missing values; d, 3 missing values; e, 4 missing values; f, 5 missing values.

* Reported for participants who immigrated to Canada (n = 135). Demographic comparisons were conducted using ANOVA, Kruskal–Wallis, χ^2^, or Fisher's exact tests, as appropriate. Cognitive and clinical comparisons were conducted using linear regression models adjusted for age, sex, and years of education. Post hoc comparisons are reported only when the omnibus *p*‐value was < 0.05.

With respect to follow‐up, to date, 99 participants have completed Year 2 remote questionnaires (33 South Asian, 34 Chinese, 32 NHW participants), with no dropouts. In addition, 46 participants have completed Year 3 in‐person follow‐up assessments (17 South Asian, 16 Chinese, and 13 NHW participants), with only four dropouts (three Chinese, one NHW).

As shown in Table 2, age, sex, years of education, and annual income did not significantly differ across South Asian, Chinese, and NHW participants. As expected, South Asian and Chinese participants were less likely to be born in Canada, and more likely to report English as a second language compared to NHW participants. Compared to Chinese participants, South Asian participants were younger at the time of immigration and had a longer duration of residence in Canada.

### 3.2 Clinical characteristics

Differences in clinical characteristics across ethnoracial groups were examined using linear regression models adjusted for age, sex, and years of education (Table 2). South Asian participants had significantly lower HDL cholesterol and higher triglyceride levels than both Chinese and NHW participants. Both South Asian and Chinese participants had higher HbA1c levels compared to NHW participants. In contrast, Chinese participants had significantly lower body mass index (BMI) than both South Asian and NHW participants. Quality of life was lower among South Asian and Chinese participants relative to NHW participants. No significant group differences were observed in depressive or anxiety symptoms, cholesterol levels (total, low‐density lipoprotein, non‐HDL), blood pressure, or waist‐to‐hip ratio.

### 3.3 Neuroimaging data

Neuroimaging analyses focused on total gray matter volume (GMV) derived from FreeSurfer version 8.0 (see Supplementary Methods for preprocessing details). After adjustment for age, sex, years of education, and estimated total intracranial volume (eTIV), linear regression models indicated that both South Asian (*β* = −0.21, *p* = 0.008) and Chinese (*β* = −0.19, *p* = 0.003) participants exhibited lower GMV relative to NHW participants, with no difference between South Asian and Chinese participants (*β* = −0.02, *p* = 0.80).

To explore potential mechanisms underlying these differences, we examined whether cardiometabolic factors that differed between South Asian, Chinese, and NHW groups mediated the association between ethnoracial group and GMV. Mediation analyses were conducted in R using the *mediation* package. Models were adjusted for age, sex, years of education, and eTIV. HbA1c significantly mediated the association between ethnoracial group and GMV. Specifically, HbA1c mediated the difference in GMV between South Asian and NHW participants (average causal mediation effect [ACME] = −0.04, *p* = 0.02) and between Chinese and NHW participants (ACME = −0.06, *p* = 0.008), accounting for approximately 21% and 34% of the total effect, respectively. In contrast, HDL cholesterol, triglycerides, and BMI did not mediate the association between ethnoracial group and GMV (Table S4).

### 3.4 Cognitive and eye tracking data

Cognitive (Table 2) and eye tracking (Table S5) outcomes were examined across ethnoracial groups using linear regression models adjusted for age, sex, and years of education. No group differences were observed in global CDR scores. On the Montreal Cognitive Assessment (MoCA), a screening measure influenced by language and cultural background,^60^ both South Asian (vs. NHW: *β* = ‐0.73, *p *< 0.001) and Chinese (vs. NHW: *β* = ‐0.66, *p *< 0.001) participants scored significantly lower than NHW participants. Scores did not differ between the two Asian groups (South Asian vs. Chinese: *β* = ‐0.07, *p *= 0.66). Results were unchanged after excluding participants with a global CDR of 0.5. Lower performance among South Asian and Chinese participants was most evident on MoCA items with greater demands on English proficiency and Western cultural knowledge (e.g., sentence repetition, verbal fluency, and animal naming). Specifically, correct responses for sentence repetition were observed in 74% of NHW participants, 29% of South Asian participants, and 9% of Chinese participants, with similar patterns for letter fluency (87% NHW, 81% South Asian, 70% Chinese) and animal naming (93% NHW, 79% South Asian, 80% Chinese)

The Cognitive Function Index (CFI) has been shown to be measurement invariant across several ethnoracial groups, including Asian and NHW individuals,^61^ indicating that it assesses subjective cognitive concerns equivalently across these groups. On the CFI, both South Asian (vs. NHW: *β* = 0.37, *p *= 0.04) and Chinese (vs. NHW: β = 0.66, *p *< 0.001) participants endorsed significantly greater cognitive concerns than NHW participants, with no difference between the two Asian groups (South Asian vs. Chinese: *β* = ‐0.29, *p *= 0.10). Results were unchanged after excluding individuals with a global CDR of 0.5.

No significant group differences were observed on the eye‐tracking measures (Table S5), including IPAST pro‐saccade reaction time, IPAST anti‐saccade reaction time and direction errors, and VPCT novelty preference scores.

## 4 DISCUSSION

Individuals of South Asian and Chinese descent are the largest and fastest‐growing ethnoracial groups in Canada,^11^ yet they are underrepresented in ADRD research.^2^, ^7^, ^8^ ^,^ The CAMERA study was designed to address this gap using a deep phenotyping approach, enabling investigation of early biological, clinical, and lifestyle factors that may influence ADRD risk and resilience.

In the current sample, demographic profiles were broadly comparable across South Asian, Chinese, and NHW participants, with no group differences observed in age, sex, or years of education. Overall, participants had high levels of education (average ∼16 years), consistent with other community‐based aging and ADRD studies.^62^, ^63^ This may reflect both recruitment bias (as individuals who participate in research are often highly educated) and features of Canadian immigration pathways that prioritize higher educational attainment (e.g., points‐based selection systems).^11^

Consistent with prior literature,^17^, ^64^ cardiometabolic risk profiles differed across ethnic groups. South Asian participants exhibited a less favorable risk profile than NHW participants, including significantly lower HDL cholesterol, higher HbA1c levels, and higher triglyceride levels. In contrast, and in line with some,^65^ but not all previous studies,^17^ Chinese participants exhibited higher HbA1c levels than NHW participants, despite having significantly lower BMI. This pattern aligns with evidence that Asian populations, including Chinese individuals, may develop insulin resistance at lower BMI thresholds than NHW individuals,^66^ potentially explaining the higher HbA1c levels observed despite lower BMI.

We further observed that both South Asian and Chinese participants had lower total GMV than NHW participants, with these differences mediated by HbA1c levels. This finding is consistent with prior cohort studies linking elevated HbA1c to markers of neurodegeneration.^67^ Together, these results suggest that glycemic dysregulation may be an important pathway contributing to brain atrophy and potentially dementia risk in South Asian and Chinese participants.

South Asian and Chinese participants endorsed significantly greater cognitive concerns on the CFI compared to NHW participants, even when analyses were restricted to clinically normal individuals (CDR = 0). Prior work has demonstrated that the CFI is measurement invariant across racial and ethnic groups, making it less likely that these differences reflect measurement bias.^61^ This pattern is consistent with a previous study in cognitively unimpaired adults that reported higher CFI scores among the two Asian subgroups relative to NHW peers.^68^ The reasons underlying greater endorsement of concerns among Asian participants will be examined in future analyses.

South Asian and Chinese participants had lower scores on the MoCA than NHW participants. These differences likely reflect measurement‐related factors rather than true differences in cognitive ability. The MoCA has not been shown to be measurement invariant in English‐speaking Asian populations, and in the present study, group differences were primarily driven by items requiring greater reliance on English proficiency and Western cultural knowledge (e.g., sentence repetition, letter fluency, animal naming). Consistent with this interpretation, prior work has shown that standard MoCA cutoffs can overestimate cognitive impairment in ethnoracially diverse adults.^69^ Together, these findings suggest that MoCA performance may not accurately capture cognitive function in diverse samples and highlight the need for assessment tools that reduce linguistic and cultural bias.

To address these concerns in the CAMERA study, we included eye‐tracking measures, which can be designed to reduce language‐dependent and culturally biased stimuli and do not require verbal responses. Although we observed no group differences on the eye‐tracking measures, formal evaluation of measurement invariance following previously established procedures^70^ is planned to confirm that these tasks operate equivalently across ethnoracial groups.

A key strength of the CAMERA study is its partnership with community stakeholders. Ongoing engagement with the CAB helps ensure that findings are relevant, interpreted within the context of participants’ lived experiences, and disseminated in ways that are meaningful and respectful to the communities involved. Participants frequently describe their involvement in CAMERA as collaborative, which may contribute to high recruitment rates (including word‐of‐mouth referrals) and sustained engagement. Many participants have expressed appreciation for receiving individualized results and for ongoing communication through quarterly newsletters and timely responses to questions. Together, these practices promote trust and sustained engagement, and have been associated with high levels of participant‐driven referrals.

To our knowledge, the CAMERA study is the only deep phenotyping study in North America designed to examine ADRD risk and resilience factors in both South Asian and Chinese communities. The Asian Cohort for Alzheimer's Disease (ACAD)^12^ represents one of the most closely related initiatives. It includes ~5,000 participants from both Canada and the U.S., and recruits individuals of Chinese, Korean, and Vietnamese ancestry. The primary aim of ACAD is to characterize genetic and lifestyle factors, with the latter mainly assessed through self‐report questionnaires. A strength of ACAD is the use of translated cognitive assessments.

Other large U.S.‐based cohort studies share similarities with CAMERA. The Multi‐Ethnic Study of Atherosclerosis (MESA)^71^ was originally designed to examine subclinical atherosclerosis in individuals of Chinese, African American, Hispanic, and NHW backgrounds. More recently, the MESA‐MIND ancillary study incorporated brain imaging and cognitive assessments, extending MESA's relevance to brain aging. The Mediators of Atherosclerosis in South Asians Living in America (MASALA)^72^ study focuses on South Asian adults and has generated rich longitudinal data on cardiovascular and metabolic risk, but does not currently include neuroimaging, ADRD biomarkers, or cognitive assessments.

Despite its many strengths, the CAMERA study has several limitations. First, all assessments are conducted in English, which may limit generalizability to individuals with limited English proficiency. This decision reflects practical and resource‐related considerations, including the availability of validated assessment tools and appropriately trained staff. Although this approach does not capture the full linguistic diversity of these populations, it reflects aspects of the Canadian context, where some immigration pathways prioritize English proficiency (e.g., points‐based selection systems). Second, although our neuropsychological test battery, including eye‐tracking tests, was designed to minimize language demands and culturally specific content, we recognize that even nonverbal cognitive tests can be influenced by cultural factors. It remains to be determined whether the eye‐tracking measures included in CAMERA are invariant across the included ethnoracial groups, and this will be formally examined in future analyses. Finally, the exclusion of individuals with major cardiovascular or cerebrovascular events, along with the relatively high education and income levels of the sample, may further limit the generalizability of our findings. In addition, bilingualism and multilingualism in some participants may contribute to cognitive reserve and will be examined more explicitly in future analyses.

Taken together, CAMERA represents an important step toward addressing the longstanding underrepresentation of South Asian and Chinese communities in ADRD research. By leveraging a deep‐phenotyping approach, the study provides a platform for identifying risk and resilience factors for ADRD and clarifying their underlying mechanisms. As the cohort expands and longitudinal data accrue, CAMERA is well positioned to generate insights that may inform future large‐scale studies and more tailored approaches to prevention and intervention for these communities.

## CONFLICT OF INTEREST STATEMENT

J.S.R. receives support from the Harquail Centre for Neuromodulation, the Dr. Sandra Black Centre for Brain Resilience & Recovery, CIHR (173253), the Alzheimer Society of Canada Research Program (New Investigator Operating Grant), and the Alzheimer's Association (AARG‐23‐1144933). MG is supported by the Canada Research Chairs program (CRC‐2021‐00374), the Harquail Centre for Neuromodulation, Sandra Black Center for Brain Resilience and Recovery, CIHR (178059), and the Alzheimer Society of Canada Research Program (ALZ23‐05). WS is supported by the Canada Research Chairs Program (CRC‐2024‐00213), the Ontario Ministry of Colleges and Universities (ER21‐16‐146), and by the Dr. Sandra Black Centre for Brain Resilience and Recovery. SEB has received personal consulting fees from Roche, Biogen, Novo Nordisk, Eisai, and Eli Lilly. She also receives peer‐reviewed research funding from the Ontario Brain Institute, Canadian Institutes of Health Research (CIHR), Leducq Foundation, Heart and Stroke Foundation of Canada, National Institutes of Health (NIH), Alzheimer's Drug Discovery Foundation, Brain Canada, Weston Brain Institute, and the Canadian Partnership for Stroke Recovery. She does not receive personal investigator fees from these funding sources. No other competing interests or funding sources were reported from the authors. Author disclosures are available in the Supporting Information.

## CONSENT STATEMENT

The CAMERA study was approved by the Research Ethics Board at Sunnybrook Health Sciences Centre (SHSC) in Toronto, Canada. All participants provided informed consent.

## Supporting information



Supporting Information

Supporting Information

## References

[1] 2025 Alzheimer's disease facts and figures. Alzheimers Dement. 2025;21(4):e70235. doi:10.1002/alz.70235

[2] Babulal GM , Quiroz YT , Albensi BC , et al. Perspectives on ethnic and racial disparities in Alzheimer's disease and related dementias: update and areas of immediate need. Alzheimers Dement. 2019;15(2):292‐312. doi:10.1016/j.jalz.2018.09.009 30555031 PMC6368893

[3] Brewster P , Barnes L , Haan M , et al. Progress and future challenges in aging and diversity research in the United States. Alzheimers Dement. 2019;15(7):995‐1003. doi:10.1016/j.jalz.2018.07.221 30240574 PMC7021489

[4] Jack CR , Andrews JS , Beach TG , et al. Revised criteria for diagnosis and staging of Alzheimer's disease: alzheimer's Association Workgroup. Alzheimer's & Dementia. 2024;20(8):5143‐5169. doi:10.1002/alz.13859 PMC11350039 38934362

[5] Lim AC , Barnes LL , Weissberger GH , et al. Quantification of race/ethnicity representation in Alzheimer's disease neuroimaging research in the USA: a systematic review. Commun Med. 2023;3(1):101. doi:10.1038/s43856-023-00333-6 37491471 PMC10368705

[6] Zhu Y , Park S , Kolady R , et al. A systematic review/meta‐analysis of prevalence and incidence rates illustrates systemic underrepresentation of individuals racialized as Asian and/or Asian‐American in ADRD research. Alzheimer's & Dementia. 2024;20(6):4315‐4330. doi:10.1002/alz.13820 PMC11180860 38708587

[7] Krishnan A , Waite LM , Stanaway FF . Representation of racial and ethnic minority groups in cohort studies evaluating risk factors for dementia: protocol for a scoping review. BMJ Open. 2021;11(5):e044404. doi:10.1136/bmjopen-2020-044404 PMC8126304 33986050

[8] Grill JD , Sperling RA , Raman R . What Should the Goals Be for Diverse Recruitment in Alzheimer Clinical Trials?. JAMA Neurol. 2022;79(11):1097‐1098. doi:10.1001/jamaneurol.2022.2274 35969392

[9] Sunderland KM , Beaton D , Arnott SR , et al. Characteristics of the Ontario Neurodegenerative Disease Research Initiative cohort. Alzheimer's & Dementia. 2023;19(1):226‐243. doi:10.1002/alz.12632 36318754

[10] Stinchcombe A , Hammond NG . Social determinants of memory change: a three‐year follow‐up of the Canadian Longitudinal Study on Aging (CLSA). Archives of Gerontology and Geriatriscs. 2023;104:104830. doi:10.1016/j.archger.2022.104830 36257162

[11] Statistics Canada . Census Profile, 2021 Census of Population. https://www12.statcan.gc.ca/census‐recensement/2021/dp‐pd/prof/index.cfm?Lang=E

[12] Ho P , Yu WH , Tee BL , et al. Asian Cohort for Alzheimer's Disease (ACAD) pilot study on genetic and non‐genetic risk factors for Alzheimer's disease among Asian Americans and Canadians. Alzheimer's & Dementia. 2024;20(3):2058‐2071. doi:10.1002/alz.13611 PMC10984480 38215053

[13] United States Census Bureau . Not All Racial and Ethnic Groups Are Aging At National Pace. 2024. https://www.census.gov/library/stories/2024/07/population‐projections.html

[14] Mayeda ER , Glymour MM , Quesenberry CP , Whitmer RA . Inequalities in dementia incidence between six racial and ethnic groups over 14 years. Alzheimer's & Dementia. 2016;12(3):216‐224. doi:10.1016/j.jalz.2015.12.007 10.1016/j.jalz.2015.12.007 PMC4969071 26874595

[15] Rabin JS , Schultz AP , Hedden T , et al. Interactive Associations of Vascular Risk and β‐Amyloid Burden With Cognitive Decline in Clinically Normal Elderly Individuals: findings From the Harvard Aging Brain Study. JAMA Neurol. 2018;75(9):1124. doi:10.1001/jamaneurol.2018.1123 29799986 10.1001/jamaneurol.2018.1123 PMC6143121

[16] Snyder HM , Corriveau RA , Craft S , et al. Vascular contributions to cognitive impairment and dementia including Alzheimer's disease. Alzheimer's & Dementia. 2015;11(6):710‐717. doi:10.1016/j.jalz.2014.10.008 10.1016/j.jalz.2014.10.008 PMC4731036 25510382

[17] Anand SS , Yusuf S , Vuksan V , et al. Differences in risk factors, atherosclerosis, and cardiovascular disease between ethnic groups in Canada: the Study of Health Assessment and Risk in Ethnic groups (SHARE). Lancet. 2000;356(9226):279‐284. doi:10.1016/s0140‐6736(00)02502‐2 11071182 10.1016/s0140-6736(00)02502-2

[18] Cardiometabolic Risk Working Group: Executive Committee , Leiter LA , Fitchett DH , et al, Cardiometabolic Risk Working Group: Executive Committee . Cardiometabolic risk in Canada: a detailed analysis and position paper by the cardiometabolic risk working group. Can J Cardiol. 2011;27(2):e1‐e33. doi:10.1016/j.cjca.2010.12.054 21459257 10.1016/j.cjca.2010.12.054

[19] Pedamallu H , Aghabazaz Z , Lancki N , et al. Prevalence and Trends in Cardiovascular Risk Factors Among Middle Aged Persons from Five Race and Ethnic Groups in the United States: a Longitudinal Analysis of Two Cohort Studies. Preprint. 2024. doi:10.1101/2024.09.27.24314520. posted online September 28. 10.1161/JAHA.124.041221 PMC13055708 41669952

[20] Chiu M , Maclagan LC , Tu JV , Shah BR . Temporal trends in cardiovascular disease risk factors among white, South Asian, Chinese and black groups in Ontario, Canada, 2001 to 2012: a population‐based study. BMJ Open. 2015;5(8):e007232. doi:10.1136/bmjopen‐2014‐007232 10.1136/bmjopen-2014-007232 PMC4538273 26260346

[21] Chiu M , Austin PC , Manuel DG , Tu JV . Comparison of cardiovascular risk profiles among ethnic groups using population health surveys between 1996 and 2007. CMAJ. 2010;182(8):E301‐310. doi:10.1503/cmaj.091676 20403888 10.1503/cmaj.091676 PMC2871219

[22] Jin K , Ding D , Gullick J , Koo F , Neubeck LA . Chinese Immigrant Paradox? Low Coronary Heart Disease Incidence but Higher Short‐Term Mortality in Western‐Dwelling Chinese Immigrants: a Systematic Review and Meta‐Analysis. JAHA. 2015;4(12):e002568. doi:10.1161/JAHA.115.002568 26683217 10.1161/JAHA.115.002568 PMC4845291

[23] Hayes‐Larson E , Zhou Y , Wu Y , et al. Estimating dementia incidence in insured older Asian Americans and Pacific Islanders in California: an application of inverse odds of selection weights. American Journal of Epidemiology. 2024;5:kwae182. doi:10.1093/aje/kwae182. Published online July. 10.1093/aje/kwae182 PMC12055469 38973744

[24] Hayes‐Larson E , Zhou Y , Wu Y , et al. Heterogeneity in the effect of type 2 diabetes on dementia incidence in a diverse cohort of Asian American and non‐Latino White older adults. American Journal of Epidemiology. 2024;193(9):1261‐1270. doi:10.1093/aje/kwae051 38949483 10.1093/aje/kwae051 PMC11369220

[25] Statistics Canada . Selected housing characteristics, low income indicators and knowledge of official languages, by visible minority and other characteristics for the population in private households. Published online 2023. doi:10.25318/4310006001‐ENG

[26] Morris JC . Clinical dementia rating: a reliable and valid diagnostic and staging measure for dementia of the Alzheimer type. Int Psychogeriatr. 1997;9(S1):173‐176. doi:10.1017/S1041610297004870 9447441 10.1017/s1041610297004870

[27] Webster KE , Wittwer JE , Feller JA . Validity of the GAITRite® walkway system for the measurement of averaged and individual step parameters of gait. Gait Posture. 2005;22(4):317‐321. doi:10.1016/j.gaitpost.2004.10.005 16274913 10.1016/j.gaitpost.2004.10.005

[28] Farhan SMK , Bartha R , Black SE , et al. The Ontario neurodegenerative disease research initiative (ONDRI). Can J Neurol Sci. 2017;44(2):196‐202. doi:10.1017/cjn.2016.415 28003035 10.1017/cjn.2016.415

[29] Smits C , Kapteyn TS , Houtgast T . Development and validation of an automatic speech‐in‐noise screening test by telephone. Int J Audiol. 2004;43(1):15‐28. doi:10.1080/14992020400050004 14974624 10.1080/14992020400050004

[30] Giguère C , Lagacé J , Ellaham NN , et al. Development of the Canadian digit triplet test in English and French. J Acoust Soc Am. 2020;147(3):EL252‐EL258. doi:10.1121/10.0000825 32237800 10.1121/10.0000825

[31] Manly JJ . Critical issues in cultural neuropsychology: profit from diversity. Neuropsychol Rev. 2008;18(3):179‐183. doi:10.1007/s11065‐008‐9068‐8 18814033 10.1007/s11065-008-9068-8 PMC2759971

[32] Vila‐Castelar C , Fox‐Fuller JT , Guzmán‐Vélez E , Schoemaker D , Quiroz YT . A cultural approach to dementia –‐ insights from US Latino and other minoritized groups. Nat Rev Neurol. 2022;18(5):307‐314. doi:10.1038/s41582‐022‐00630‐z 35260817 10.1038/s41582-022-00630-z PMC9113534

[33] Lee H , Tzuang M , Chow TW , et al. Translation and cultural adaptation of tools to assess diverse Asian American and Asian Canadian subgroups: the Asian Cohort for Alzheimer's disease (ACAD) study. Alzheimers Dement. 2025;21(6):e70311. doi:10.1002/alz.70311 40528300 10.1002/alz.70311 PMC12173839

[34] Malik HB , Norman JB . Best practices and methodological strategies for addressing generalizability in neuropsychological assessment. J Pediatr Neuropsychol. 2023;9(2):47‐63. doi:10.1007/s40817‐023‐00145‐5 37250805 10.1007/s40817-023-00145-5 PMC10182845

[35] Kano F , Hirata S . Great apes make anticipatory looks based on long‐term memory of single events. Curr Biol. 2015;25(19):2513‐2517. doi:10.1016/j.cub.2015.08.004 26387711 10.1016/j.cub.2015.08.004

[36] Kano F , Tomonaga M . How chimpanzees look at pictures: a comparative eye‐tracking study. Proc R Soc B. 2009;276:1949‐1955. doi:10.1098/rspb.2008.1811 10.1098/rspb.2008.1811 PMC2677242 19324790

[37] Wilson VAD , Zuberbühler K , Bickel B . The evolutionary origins of syntax: event cognition in nonhuman primates. Sci Adv. 2022;8(25):eabn8464. doi:10.1126/sciadv.abn8464 35731868 10.1126/sciadv.abn8464 PMC9216513

[38] Werchan DM , Hume A , Zhang M , Vo T , Brito NH . From focus to function: longitudinal insights into infant attention and emerging executive functions via remote webcam eye tracking. Dev Psychol. 2025;61(5):957‐963. doi:10.1037/dev0001948 40111879 10.1037/dev0001948 PMC12039938

[39] Horváth K , Hannon B , Ujma PP , Gombos F , Plunkett K . Memory in 3‐month‐old infants benefits from a short nap. Develop Sci. 2018;21(3):e12587. doi:10.1111/desc.12587 10.1111/desc.12587 28722249

[40] Richmond JL , Zhao JL , Burns MA . What goes where? Eye tracking reveals spatial relational memory during infancy. J Exp Child Psychol. 2015;130:79‐91. doi:10.1016/j.jecp.2014.09.013 25462033 10.1016/j.jecp.2014.09.013

[41] Sharma S , Kim H , Harris H , Haberstroh A , Wright HH , Rothermich K . Eye tracking measures for studying language comprehension deficits in aphasia: a systematic search and scoping review. J Speech Lang Hear Res. 2021;64(3):1008‐1022. doi:10.1044/2020_JSLHR‐20‐00287 33606952 10.1044/2020_JSLHR-20-00287

[42] Proudfoot M , Menke RAL , Sharma R , et al. Eye‐tracking in amyotrophic lateral sclerosis: a longitudinal study of saccadic and cognitive tasks. Amyotroph Lateral Scler Frontotemporal Degener. 2015;17(1‐2):101‐111. doi:10.3109/21678421.2015.1054292 26312652 10.3109/21678421.2015.1054292 PMC5127416

[43] Coe BC , Munoz DP . Mechanisms of saccade suppression revealed in the anti‐saccade task. Phil Trans R Soc B. 2017;372(1718):20160192. doi:10.1098/rstb.2016.0192 28242726 10.1098/rstb.2016.0192 PMC5332851

[44] Munoz DP , Everling S . Look away: the anti‐saccade task and the voluntary control of eye movement. Nat Rev Neurosci. 2004;5(3):218‐228. doi:10.1038/nrn1345 14976521 10.1038/nrn1345

[45] Manns JR , Stark CEL , Squire LR . The visual paired‐comparison task as a measure of declarative memory. Proc Natl Acad Sci USA. 2000;97(22):12375‐12379. doi:10.1073/pnas.220398097 11027310 10.1073/pnas.220398097 PMC17349

[46] McDowell JE , Dyckman KA , Austin BP , Clementz BA . Neurophysiology and neuroanatomy of reflexive and volitional saccades: evidence from studies of humans. Brain and Cognition. 2008;68(3):255‐270. doi:10.1016/j.bandc.2008.08.016 18835656 10.1016/j.bandc.2008.08.016 PMC2614688

[47] Fernandez‐Ruiz J , Peltsch A , Alahyane N , et al. Age related prefrontal compensatory mechanisms for inhibitory control in the antisaccade task. NeuroImage. 2018;165:92‐101. doi:10.1016/j.neuroimage.2017.10.001 28988829 10.1016/j.neuroimage.2017.10.001

[48] Peltsch A , Hemraj A , Garcia A , Munoz DP . Age‐related trends in saccade characteristics among the elderly. Neurobiology of Aging. 2011;32(4):669‐679. doi:10.1016/j.neurobiolaging.2009.04.001 19414208 10.1016/j.neurobiolaging.2009.04.001

[49] Yep R , Smorenburg ML , Riek HC , et al. Interleaved Pro/Anti‐saccade Behavior Across the Lifespan. Front Aging Neurosci. 2022;14:842549. doi:10.3389/fnagi.2022.842549 35663573 10.3389/fnagi.2022.842549 PMC9159803

[50] Peltsch A , Hemraj A , Garcia A , Munoz DP . Saccade deficits in amnestic mild cognitive impairment resemble mild Alzheimer's disease. Eur J of Neuroscience. 2014;39(11):2000‐2013. doi:10.1111/ejn.12617 10.1111/ejn.12617 24890471

[51] Riek HC , Brien DC , Coe BC , et al. Cognitive correlates of antisaccade behaviour across multiple neurodegenerative diseases. Brain Communications. 2023;5(2):fcad049. doi:10.1093/braincomms/fcad049 36970045 10.1093/braincomms/fcad049 PMC10036290

[52] Crutcher MD , Calhoun‐Haney R , Manzanares CM , Lah JJ , Levey AI , Zola SM . Eye Tracking During a Visual Paired Comparison Task as a Predictor of Early Dementia. Am J Alzheimers Dis Other Demen. 2009;24(3):258‐266. doi:10.1177/1533317509332093 19246573 10.1177/1533317509332093 PMC2701976

[53] Van Der Cruyssen I , Ben‐Shakhar G , Pertzov Y , et al. The validation of online webcam‐based eye‐tracking: the replication of the cascade effect, the novelty preference, and the visual world paradigm. Behav Res. 2023;56(5):4836‐4849. doi:10.3758/s13428‐023‐02221‐2 10.3758/s13428-023-02221-2 PMC11289066 37648844

[54] Whitehead JC , Li L , McQuiggan DA , Gambino SA , Binns MA , Ryan JD . Portable eyetracking‐based assessment of memory decline. Journal of Clinical and Experimental Neuropsychology. 2018;40(9):904‐916. doi:10.1080/13803395.2018.1444737 29547067 10.1080/13803395.2018.1444737

[55] Hannula DE . Worth a glance: using eye movements to investigate the cognitive neuroscience of memory. Front Hum Neurosci. 2010;4. doi:10.3389/fnhum.2010.00166 10.3389/fnhum.2010.00166 PMC2995997 21151363

[56] Zola SM , Manzanares CM , Clopton P , Lah JJ , Levey AI . A Behavioral Task Predicts Conversion to Mild Cognitive Impairment and Alzheimer's Disease. Am J Alzheimers Dis Other Demen. 2013;28(2):179‐184. doi:10.1177/1533317512470484 23271330 10.1177/1533317512470484 PMC3670591

[57] Pavey TG , Gomersall SR , Clark BK , Brown WJ . The validity of the GENEActiv wrist‐worn accelerometer for measuring adult sedentary time in free living. Journal of Science and Medicine in Sport. 2016;19(5):395‐399. doi:10.1016/j.jsams.2015.04.007 25956687 10.1016/j.jsams.2015.04.007

[58] Sano M , Egelko S , Zhu CW , et al. Participant satisfaction with dementia prevention research: results from Home‐Based Assessment trial. Alzheimer's & Dementia. 2018;14(11):1397‐1405. doi:10.1016/j.jalz.2018.05.016 10.1016/j.jalz.2018.05.016 30297140

[59] Roberts JS , Ferber R , Blacker D , Rumbaugh M , Grill JD , for the Advisory Group on Risk Evidence Education for Dementia (AGREED) . Disclosure of individual research results at federally funded Alzheimer's Disease Research Centers. A&D Transl Res & Clin Interv. 2021;7(1):e12213. doi:10.1002/trc2.12213 10.1002/trc2.12213 PMC8515553 34692986

[60] O'Driscoll C , Shaikh M . Cross‐Cultural Applicability of the Montreal Cognitive Assessment (MoCA): a Systematic Review. JAD. 2017;58(3):789‐801. doi:10.3233/JAD‐161042. Chopard G, ed. 28482634 10.3233/JAD-161042

[61] Ruthirakuhan M , Wood Alexander M , Cogo‐Moreira H , et al. Investigating the factor structure of the preclinical Alzheimer cognitive composite and cognitive function index across racial/ethnic, sex, and Aβ status groups in the A4 study. J Prevent Alzheimer's Dis. 2024;11(1):48‐55. doi:10.14283/jpad.2023.98 10.14283/jpad.2023.98 38230716

[62] Dagley A , LaPoint M , Huijbers W , et al. Harvard aging brain study: dataset and accessibility. Neuroimage. 2017;144(Pt B):255‐258. doi:10.1016/j.neuroimage.2015.03.069 25843019 10.1016/j.neuroimage.2015.03.069 PMC4592689

[63] Petersen RC , Aisen PS , Beckett LA , et al. Alzheimer's disease neuroimaging initiative (ADNI): clinical characterization. Neurology. 2010;74(3):201‐209. doi:10.1212/WNL.0b013e3181cb3e25 20042704 10.1212/WNL.0b013e3181cb3e25 PMC2809036

[64] Gupta M , Brister S . Is South Asian ethnicity an independent cardiovascular risk factor?. Can J Cardiol. 2006;22(3):193‐197. doi:10.1016/s0828‐282x(06)70895‐9 16520847 10.1016/s0828-282x(06)70895-9 PMC2528919

[65] Alangh A , Chiu M , Shah BR . Rapid increase in diabetes incidence among Chinese Canadians between 1996 and 2005. Diabetes Care. 2013;36(10):3015‐3017. doi:10.2337/dc13‐0052 23723356 10.2337/dc13-0052 PMC3781544

[66] Chiu KC , Cohan P , Lee NP , Chuang LM . Insulin sensitivity differs among ethnic groups with a compensatory response in beta‐cell function. Diabetes Care. 2000;23(9):1353‐1358. doi:10.2337/diacare.23.9.1353 10977032 10.2337/diacare.23.9.1353

[67] Byun MS , Kim HJ , Yi D , et al. Differential effects of blood insulin and HbA1c on cerebral amyloid burden and neurodegeneration in nondiabetic cognitively normal older adults. Neurobiology of Aging. 2017;59:15‐21. doi:10.1016/j.neurobiolaging.2017.07.004 28780367 10.1016/j.neurobiolaging.2017.07.004

[68] Robinson T , Klinger H , Buckley R , et al. Subjective cognitive decline across ethnoracial groups in the A4 study. Alzheimers Dement. 2023;19(9):4084‐4093. doi:10.1002/alz.13138 37218387 10.1002/alz.13138 PMC10524317

[69] Milani SA , Marsiske M , Cottler LB , Chen X , Striley CW . Optimal cutoffs for the Montreal cognitive assessment vary by race and ethnicity. Alz Dem Diag Ass & Dis Mo. 2018;10(1):773‐781. doi:10.1016/j.dadm.2018.09.003 10.1016/j.dadm.2018.09.003 PMC6247398 30505927

[70] Avila JF , Rentería MA , Witkiewitz K , Verney SP , Vonk JMJ , Manly JJ . Measurement invariance of neuropsychological measures of cognitive aging across race/ethnicity by sex/gender groups. Neuropsychology. 2020;34(1):3‐14. doi:10.1037/neu0000584 31464473 10.1037/neu0000584 PMC8377699

[71] Bild DE , Bluemke DA , Burke GL , et al. Multi‐ethnic study of atherosclerosis: objectives and design. Am J Epidemiol. 2002;156(9):871‐881. doi:10.1093/aje/kwf113 12397006 10.1093/aje/kwf113

[72] Kanaya AM , Kandula N , Herrington D , et al. Mediators of atherosclerosis in South Asians Living in America (MASALA) study: objectives, methods, and cohort description. Clin Cardiol. 2013;36(12):713‐720. doi:10.1002/clc.22219 24194499 10.1002/clc.22219 PMC3947423

